# Asbestos: use, bans and disease burden in Europe

**DOI:** 10.2471/BLT.13.132118

**Published:** 2014-09-17

**Authors:** Takashi Kameda, Ken Takahashi, Rokho Kim, Ying Jiang, Mehrnoosh Movahed, Eun-Kee Park, Jorma Rantanen

**Affiliations:** a Department of Environmental Epidemiology, Institute of Industrial Ecological Sciences, University of Occupational and Environmental Health, Iseigaoka 1-1, Yahatanishiku, Kitakyushu, Japan.; b WHO Western Pacific Regional Office, Suva, Fiji.; c Department of Medical Humanities and Social Medicine, Kosin University College of Medicine, Busan, Republic of Korea.; d University of Jyväskylä, Jyväskylä, Finland.

## Abstract

**Objective:**

To analyse national data on asbestos use and related diseases in the European Region of the World Health Organization (WHO).

**Methods:**

For each of the 53 countries, per capita asbestos use (kg/capita/year) and age-adjusted mortality rates (deaths/million persons/year) due to mesothelioma and asbestosis were calculated using the databases of the United States Geological Survey and WHO, respectively. Countries were further categorized by ban status: early-ban (ban adopted by 2000, *n* = 17), late-ban (ban adopted 2001–2013, *n* = 17), and no-ban (*n* = 19).

**Findings:**

Between 1920–2012, the highest per capita asbestos use was found in the no-ban group. After 2000, early-ban and late-ban groups reduced their asbestos use levels to less than or equal to 0.1 kg/capita/year, respectively, while the no-ban group maintained a very high use at 2.2 kg/capita/year. Between 1994 and 2010, the European Region registered 106 180 deaths from mesothelioma and asbestosis, accounting for 60% of such deaths worldwide. In the early-ban and late-ban groups, 16/17 and 15/17 countries, respectively, reported mesothelioma data to WHO, while only 6/19 countries in the no-ban group reported such data. The age-adjusted mortality rates for mesothelioma for the early-ban, late-ban and no-ban groups were 9.4, 3.7 and 3.2 deaths/million persons/year, respectively. Asbestosis rates for the groups were 0.8, 0.9 and 1.5 deaths/million persons/year, respectively.

**Conclusion:**

Within the European Region, the early-ban countries reported most of the current asbestos-related deaths. However, this might shift to the no-ban countries, since the disease burden will likely increase in these countries due the heavy use of asbestos.

## Résumé

**Objectif:**

Analyser les données nationales sur l'utilisation de l'amiante et les maladies qui y sont associées, dans la région européenne de l'Organisation mondiale de la Santé (OMS).

**Méthodes:**

Pour chacun des 53 pays, l'utilisation de l'amiante par habitant (kg/habitant/an) et les taux de mortalité selon l'âge (décès/millions de personnes/an) dus au mésothéliome et à l'asbestose ont été calculés en utilisant les bases de données de l'USGS (United States Geological Survey) et de l'OMS, respectivement. Les pays ont ensuite été classés en fonction de leur statut d'interdiction: interdiction précoce (interdiction adoptée avant 2000, n = 17), interdiction tardive (interdiction adoptée entre 2001–2013, n = 17) et aucune interdiction (n = 19).

**Résultats:**

Entre 1920 et 2012, l'utilisation la plus élevée d'amiante par habitant a été trouvée dans le groupe sans aucune interdiction. Après 2000, les groupes à interdiction précoce et à interdiction tardive ont réduit leurs niveaux d'utilisation de l'amiante à un niveau inférieur ou égal à 0,1 kg/habitant/an, respectivement, tandis que le groupe sans aucune interdiction a maintenu une utilisation très élevée de 2,2 kg/habitant/an. Entre 1994 et 2010, la région européenne a déclaré 106 180 décès causés par le mésothéliome et l'asbestose, représentant 60% de ces causes de décès dans le monde. Dans les groupes à interdiction précoce et à interdiction tardive, 16/17 et 15/17 pays, respectivement, ont fourni des données relatives au mésothéliome à l'OMS, alors que seuls 6/19 pays dans le groupe sans aucune interdiction ont fourni de telles données. Les taux de mortalité selon l'âge du mésothéliome pour les groupes à interdiction précoce, à interdiction tardive et sans aucune interdiction étaient de 9,4, 3,7 et 3,2 décès/million de personnes/an, respectivement. Les taux d'asbestose des mêmes groupes étaient de 0,8, 0,9 et 1,5 décès/million de personnes/an, respectivement.

**Conclusion:**

Au sein de la région européenne, les pays à interdiction précoce ont signalé la plupart des décès actuels liés à l'asbestose. Cependant, cela pourrait se déplacer vers les pays sans aucune interdiction, puisque la charge de morbidité augmentera probablement dans ces pays en raison de leur utilisation importante de l'amiante.

## Resumen

**Objetivo:**

Analizar los datos nacionales sobre el uso del amianto y las enfermedades relacionadas a este en la región europea de la Organización Mundial de la Salud (OMS).

**Métodos:**

Para cada uno de los 53 países, se calculó el uso del amianto por cápita (kg/cápita/año) y las tasas de mortalidad ajustadas por edad (muertes/millón de personas/año) causadas por mesotelioma y asbestosis a través de, respectivamente, las bases de datos de la Encuesta Geológica de los Estados Unidos y la OMS. Además, los países se clasificaron según el estado de prohibición: prohibición temprana (prohibición adoptada en el año 2000, n = 17), prohibición tardía (prohibición adoptada entre 2001–2013, n = 17) y sin prohibición (n = 19).

**Resultados:**

El grupo sin prohibición presentó el mayor uso de amianto por cápita entre 1920–2012. A partir del año 2000, los grupos con prohibiciones tempranas y tardías redujeron sus niveles de uso de amianto a menos de o igual a 0,1 kg/cápita/año, respectivamente, mientras que el grupo sin prohibición mantuvo un uso muy elevado de 2,2 kg/cápita/año. Entre 1994 y 2010, la Región de Europa registró 106 180 muertes por mesotelioma y asbestosis, lo que representa el 60 % de este tipo de muertes en todo el mundo. Los grupos con prohibiciones tempranas y tardías, 16/17 y 15/17 países respectivamente, comunicaron los datos de mesotelioma a la OMS, mientras que solo 6/19 países del grupo sin prohibición facilitaron dicha información. Las tasas de mortalidad ajustadas por edad para el mesotelioma en los países con prohibiciones tempranas y tardías y sin prohibición fueron de 9,4, 3,7 y 3,2 muertes/millón de personas/año, respectivamente, y las tasas de asbestosis, de 0,8, 0,9 y 1,5 muertes/millón de personas/año, respectivamente.

**Conclusión:**

En la Región de Europa, los países con la prohibición temprana notificaron el mayor número de las muertes actuales relacionadas con el amianto. Sin embargo, esto podría cambiar a los países sin prohibición, ya que es probable que la carga de la enfermedad aumente en dichos países debido al gran uso de amianto.

## ملخص

**الغرض:**

تحليل البيانات الوطنية بشأن استخدام الحرير الصخري (الأسبستوس) والأمراض ذات الصلة في الإقليم الأوروبي لمنظمة الصحة العالمية.

**الطريقة:**

في كل بلد من البلدان الثلاثة والخمسين، تم حساب نصيب الفرد من استخدام الحرير الصخري (الأسبستوس) (كلغ/فرد/سنة) ومعدلات الوفيات المصححة باحتساب العمر (وفاة/مليون نسمة/سنة) الناجمة عن ورم المتوسطة وداء الأسبستوس، باستخدام قواعد بيانات المسح الجيولوجي للولايات المتحدة ومنظمة الصحة العالمية، على التوالي. وتم تصنيف البلدان بعد ذلك حسب حالة الحظر: الحظر المبكر (الحظر الذي تم اعتماده بحلول عام 2000، العدد = 17)، الحظر المتأخر (الحظر الذي تم اعتماده في الفترة من 2001 إلى 2013، العدد = 17)، وعدم الحظر (العدد = 19).

**النتائج:**

بين عامي 1920 و2012، تبين وجود أعلى نصيب للفرد من استخدام الحرير الصخري (الأسبستوس) في مجموعة عدم الحظر. وبعد عام 2000، قللت مجموعتا الحظر المبكر والحظر المتأخر مستويات استخدامهما للحرير الصخري (الأسبستوس) إلى أقل من أو يساوي 0.1 كلغ/فرد/سنة، على التوالي، بينما حافظت مجموعة عدم الحظر على استخدام عال جداً بمعدل 2.2 كلغ/فرد/سنة. وبين عامي 1994 و2010، سجل الإقليم الأوروبي 106180 حالة وفاة ناجمة عن ورم المتوسطة وداء الأسبستوس، وهو يمثل 60 % من حالات الوفاة على الصعيد العالمي. وقام 16 من بين 17 بلداً و15 من بين 17 بلداً على التوالي في مجموعتي الحظر المبكر والحظر المتأخر بالإبلاغ عن بيانات ورم المتوسطة إلى منظمة الصحة العالمية في حين لم يقم بالإبلاغ عن هذه البيانات إلا 6 من بين 19 بلداً في مجموعة عدم الحظر. وكانت معدلات الوفيات المصححة بحسب العمر في ورم المتوسطة بالنسبة لمجموعات الحظر المبكر والحظر المتأخر وعدم الحظر 9.4 و3.7 و3.2 وفاة/مليون نسمة/سنة، على التوالي. وكانت معدلات داء الأسبستوس لدى المجموعات 0.8 و0.9 و1.5 وفاة/مليون نسمة/سنة، على التوالي.

**الاستنتاج:**

أبلغت بلدان الحظر المبكر داخل الإقليم الأوروبي عن معظم حالات الوفاة الراهنة ذات الصلة بالحرير الصخري (الأسبستوس). إلا أنه من المحتمل أن يتحول هذا الأمر إلى بلدان عدم الحظر، حيث يرجح ازدياد عبء المرض في هذه البلدان بسبب استخدام الحرير الصخري (الأسبستوس) على نحو كثيف.

## 摘要

**目的:**

分析世界卫生组织（WHO）欧洲地区有关石棉使用国家数据和相关疾病。

**方法:**

对于53个国家中的每个国家，分别使用美国地质调查局和WHO的数据库计算人均石棉使用（公斤/人/年）以及间皮瘤和石棉肺年龄调整死亡率（例死亡/百万人/年）。按禁令状态将国家进一步分类：较早禁用（2000年之前禁止，*n* = 17），较晚禁用（2001–2013年禁止，*n* = 17），和未禁用（*n* = 19）。

**结果:**

在1920–2012年之间，在未禁用组中发现人均石棉使用量最高。2000年之后，较早禁用和较晚禁用组石棉用量水平有所减少，分别降至小于或等于0.1公斤/人/年，而未禁用组保持着非常高的使用量，为2.2公斤/人/年。从1994年到2010年，欧洲区域登记106 180人死于间皮瘤和石棉肺，占全球同类死亡人数的60%。在较早禁用和较晚禁用组中，16/17和15/17个国家分别向WHO报告间皮瘤数据，而未禁用组中只有6/19的国家公布这些数据。较早禁用、较晚禁用和未禁用组间皮瘤年龄调整死亡率分别为9.4、3.7和3.2例死亡/百万人/年。各个组的石棉肺死亡率分别为0.8、0.9和1.5例死亡/百万人/年。

**结论:**

在欧洲地区，较早禁用国家是当前石棉相关的死亡大多数案例的报告来源。然而，这种情况可能转移到未禁用国家，因为这些国家的疾病负担可能会由于大量使用石棉而增加

## Резюме

**Цель:**

Проанализировать национальные данные Всемирной организации здравоохранения (ВОЗ) об использовании асбеста и связанных с ним заболеваний в Европейском регионе.

**Методы:**

Для каждой из 53 стран были рассчитаны уровни использования асбеста на душу населения (кг/на душу населения/год) и смертности, скорректированной по возрасту (смертей/млн человек/год), от мезотелиомы и асбестоза, используя базы данных Геологической службы США и ВОЗ соответственно. Далее страны были дополнительно классифицированы по статусу принятия запрета на использование асбеста: ранний запрет (принятый к 2000 г., *n* = 17), поздний запрет (принятый между 2001—2013 гг., *n* = 17) и отсутствие запрета (*n* = 19).

**Результаты:**

За период 1920—2012 гг. самый высокий уровень использования асбеста на душу населения был зафиксирован в группе стран, где запрет отсутствует. После 2000 года страны с ранним и поздним принятием запрета сократили свои уровни использования асбеста до 0,1 кг/чел/год, в то время как в группе стран, не имеющих запрета, сохранялся очень высокий уровень использования, равный 2,2 кг/чел/год. В период с 1994 по 2010 гг. в Европейском регионе было зарегистрировано 106 180 смертей от мезотелиомы и асбестоза, что составляет 60% от таких смертей в мире. В группе стран с ранним и поздним запретом 16 из 17 и 15 из 17 стран, соответственно, сообщили в ВОЗ данные о заболеваемости мезотелиомой, в то время как в группе, не имеющей запрета, только 6 из 19 стран сообщили такие данные. Показатели смертности от мезотелиомы, скорректированные по возрасту, среди стран с ранним запретом, поздним запретом и отсутствием такового составили 9,4, 3,7 и 3,2 смертей/млн. человек/год соответственно. Уровни асбестоза для этих групп составили 0,8, 0,9 и 1,5 смертей/млн. человек/год соответственно.

**Вывод:**

В Европейском регионе страны с ранним запретом сообщили о большинстве смертей, связанных с асбестом. Подобные показатели, тем не менее, могут распространиться на группу стран с отсутствием запрета, так как, вероятно, что в связи с интенсивным использованием асбеста бремя болезней в этих странах увеличится.

## Introduction

The World Health Organization (WHO), joined by the International Labour Organization (ILO)^1^ and the United Nations Environment Programme, has called on countries throughout the world to eliminate asbestos-related diseases.^2^^–^^4^ WHO advises that the best way to eliminate such diseases is to stop using all types of asbestos.^2^ Although numerous countries have adopted national asbestos bans, many others continue to use asbestos at various levels. The use has declined 55% from its historical peak of 4.7 million metric tonnes per year in 1980,^5^ but more than 2 million metric tonnes per year are still used worldwide.^6^^,^^7^

WHO estimates that 107 000 global annual deaths are caused by mesothelioma, asbestos-related lung cancer and asbestosis.^8^ In 2005, occupational exposure to asbestos was estimated to cause 43 000 mesothelioma deaths^9^ and 7000 deaths due to asbestosis^10^^,^^11^ worldwide. Of those caused by mesothelioma, 7000 were attributed to Europe.^9^ However, the current and future burden of asbestos-related diseases in Europe has not been fully addressed, nor have such data been examined in relation to national asbestos bans.

Through the Parma Declaration on Environment and Health, member countries of the WHO Regional Office for Europe agreed on the need to eliminate asbestos-related diseases.^12^ WHO and ILO specifically urged each country to formulate a national programme for eliminating asbestos-related diseases and develop a national asbestos profile^3^ as milestones for implementing the Parma Declaration by 2015.^12^ Asbestos use is a key item of a national asbestos profile. The volume of asbestos produced per person has been used to characterize the asbestos situation in various populations,^13^^–^^15^ and can serve as a surrogate for population-level exposure. In addition, a list of asbestos use per capita across countries and over time^16^ has been included in a recent monograph of the International Agency for Research on Cancer.^17^ Per capita asbestos use has also been employed to estimate and predict asbestos-related diseases in different populations.^16^^,^^18^^–^^20^

We conducted a descriptive analysis of national data on asbestos use and asbestos-related diseases in Europe accounting for the status of national asbestos bans.

## Methods

European countries were defined as the 53 countries in the European Region of WHO. Data on raw asbestos in these countries were obtained from the database of the United States Geological Survey^5^^,^^6^ and its updated data file (RL Virta, United States Geological Survey, personal communication, March 6, 2013). The definition of use – production plus import minus export – followed that of the United States Geological Survey.^5^ Data on asbestos use by country were available in 10-year intervals for 1920–1970, in 5-year intervals for 1970–1995 and annually for 1995–2012. We treated a reported negative value of asbestos use (e.g. reflecting storage from previous years) as zero. For years lacking data, reported data from the closest years were interpolated. We also retrieved information on the national status of asbestos bans^21^^,^^22^, ratification of the ILO Asbestos Convention^23^ and health system ranking.^24^

Data on asbestos-related diseases were extracted from the WHO mortality database;^25^ these included the number of deaths recorded as mesothelioma (*International Classification of Diseases*, 10th Revision [ICD-10], C45)^26^ or any subcategory and asbestosis (ICD-10, J61) between 1994 and 2010. Asbestos-related lung cancers were precluded from our analysis because of difficulties in attributing causation. Separately, deaths recorded for malignant neoplasm of the pleura (ICD-9, 163), a condition generally synonymous with mesothelioma of the pleura, were counted between 1994 and 2009. To investigate countries that did not report data to WHO, we searched PubMed, governmental websites and other websites that we thought were credible^27^^–^^30^ for national frequency data on asbestos use and asbestos-related diseases. To calculate rates, national population data for 1920–2012 were obtained in the following order – depending on data availability and reliability – from WHO,^25^ the United States Census Bureau^31^ or Lahmeyer.^32^

To analyse asbestos use and asbestos-related diseases in each country, we calculated per capita asbestos use (kg/capita/year) and age-adjusted mortality rates (deaths/million persons/year), respectively. Age-adjusted mortality rates were calculated using a direct age-adjustment method with reference to the WHO world standard population.^33^ We analysed all countries individually, together and in groups based on national asbestos ban status, i.e. early-ban (ban adopted by 2000; *n* = 17 countries), late-ban (ban adopted 2001–2013; *n* = 17 countries), and no-ban (no ban adopted as of 2013; *n* = 19 countries).

To provide continuity with data from currently existing countries, data on historical asbestos use from countries that had undergone political transitions (e.g. dissolution or unification) or had been combined with other countries or entities by the United States Geological Survey (*n* = 14) were obtained as follows. First, in the United States Geological Survey database, data for the Soviet Union (1920–1990) represented Kazakhstan and the Russian Federation combined. We apportioned the data between Kazakhstan and the Russian Federation according to the ratio of use recorded by these countries between 1995 and 2012. Second, data for West and East Germany (1950–1985) were combined into Germany. Third, data for Czechoslovakia (1920–1990) were apportioned to the ratio of asbestos use recorded by the Czech Republic and Slovakia between 1995 and 2012. Similarly, the data for Montenegro and Serbia (1930–1990) were apportioned to the ratios recorded by Bosnia and Herzegovina, Croatia, Montenegro, Serbia, Slovenia and the former Yugoslav Republic of Macedonia between 1995 and 2012. In the United States Geological Survey database, the data for Montenegro and Serbia (1999–2005) were combined; we apportioned the data to Montenegro and Serbia according to the sizes of the respective populations during this period. Finally, the combined data for Belgium and Luxembourg (1930–2005) were similarly apportioned to Belgium and Luxembourg.

To exploit all available data, we assessed asbestos use from 1920–2012 and asbestos-related disease mortality from 1994 to 2010 (the disease category for mesothelioma was included in the WHO mortality database in 1994). The period for asbestos use was divided into 1920–1970, 1971–2000 and 2001–2012. An early cut-off point – 1970 – was chosen to allow a sufficient interval for observation of asbestos-related diseases and to be coherent with our previous studies.^16^^,^^18^^,^^34^^,^^35^ A later cut-off point – 2000 – was used to separate recent asbestos trends in both use and related diseases.

Based on our earlier finding that 1.0 kg/capita/year of asbestos use corresponded to 2.4 and 1.6-fold increases in mesothelioma deaths among men and women, respectively,^18^ we considered this level to be high. Asbestos use of 2.0 kg/capita/year was considered very high. For asbestos-related diseases, we considered age-adjusted mortality rate levels for mesothelioma and asbestosis exceeding those of the world average (5.2 and 0.8 deaths/million persons/year, respectively) to be high.

All data were compiled using Microsoft Excel (Microsoft Corporation, Redmond, United States of America). Age-adjusted mortality rates were calculated using SAS Version 9.1 (SAS Institute, Inc., Cary, USA).

## Results

Andorra, Monaco and San Marino did not report any data for any of the indicators during the whole study period. Table 1 shows asbestos use and related disease mortality and the national asbestos ban status for each country. From 1920–1970, six countries recorded very high levels of asbestos use: Belgium, Cyprus, Denmark, Israel, Kazakhstan and Luxembourg. An additional 11 countries recorded high levels. From 1971–2000, the number of countries recording high and very high levels of use increased to 14 and 13, respectively. Between 2001 and 2012, most countries in Europe reduced their use, including those that previously used very high or high levels of asbestos: 43 countries used less than 0.5 kg/capita/year, of which 36 countries used less than 0.1 kg/capita/year. In contrast, Kazakhstan, Kyrgyzstan and the Russian Federation recorded very high levels and Belarus, Ukraine and Uzbekistan reported high levels.

**Table 1:** Status of asbestos bans, asbestos use and related diseases, Europe^a^, 1920–2012

Country	Status of asbestos ban^b^	Average per capita asbestos use, kg/capita/year^c^				Age-adjusted mortality rate, per million people (no. of reported years)^d^	
		1920–1970	1971–2000	2001–2012		Mesothelioma^e^	Asbestosis^f^
Albania	None	–	0.37	0.00		–	–
Andorra	None	–	–	–		–	–
Armenia	None	–	0.13	0.10		–	–
Austria	Early	1.17	2.09	0.00		6.35 (9)	0.42 (8)
Azerbaijan	None	–	0.39	0.41		–	–
Belarus	None	–	0.85	1.86		–	–
Belgium	Early	3.08	3.02	0.00		9.34 (5)	1.26 (5)
Bosnia and Herzegovina	None	0.00	0.01	0.00		–	–
Bulgaria	Late	0.14	1.31	0.02		1.21 (6)	1.41 (6)
Croatia	Late	0.78	3.57	0.39		7.58 (16)	0.94 (11)
Cyprus	Late	6.41	2.36	0.01		7.72 (7)	0.73 (1)
Czech Republic	Late	0.82	1.85	0.06		3.12 (17)	0.42 (12)
Denmark	Early	2.16	1.97	0.00		8.87 (13)	1.91 (13)
Estonia	Late	0.07	0.06	0.26		5.78 (14)	0.44 (1)
Finland	Early	1.49	0.86	0.03		8.96 (15)	2.39 (15)
France	Early	1.08	1.44	0.00		7.74 (10)	0.79 (10)
Georgia	None	–	0.00	0.01		1.49 (8)	0.82 (2)
Germany	Early	1.17	2.18	0.00		7.04 (13)	0.71 (13)
Greece	Late	0.41	1.28	0.00		–	–
Hungary	Late	0.78	2.36	0.03		3.01 (14)	0.24 (6)
Iceland	Early	1.29	0.30	0.01		24.58 (13)	4.59 (2)
Ireland	Early	–	1.57	0.19		5.77 (4)	0.96 (3)
Israel	Early	3.19	0.56	0.01		4.72 (12)	0.43 (6)
Italy	Early	0.83	1.61	0.00		10.37 (5)	0.30 (5)
Kazakhstan	None	6.09	18.88	8.47		–	–
Kyrgyzstan	None	–	3.12	2.72		2.56 (7)	–
Latvia	Late	0.26	0.66	0.08		5.68 (15)	–
Lithuania	Late	0.05	0.14	0.00		3.53 (13)	–
Luxembourg	Late	3.48	3.13	0.08		13.59 (12)	2.45 (2)
Malta	Late	–	–	0.00		21.33 (15)	6.31 (7)
Monaco	None	–	–	–		–	–
Montenegro	None	0.35	0.95	0.02		5.31 (6)	0.81(3)
Netherlands	Early	0.84	0.87	0.00		15.91 (15)	0.49 (15)
Norway	Early	0.98	0.36	0.00		7.99 (15)	2.07 (15)
Poland	Early	0.39	1.72	0.00		2.19 (12)	0.16 (12)
Portugal	Late	0.27	1.06	0.11		1.97 (6)	0.19 (4)
Republic of Moldova	None	–	0.84	0.06		4.20 (15)	–
Romania	Late	0.62	0.76	0.24		2.19 (12)	0.12 (5)
Russian Federation	None	1.53	7.86	2.26		–	–
San Marino	None	–	–	–		–	–
Serbia	Late	0.25	0.80	0.01		2.99 (14)	0.50 (3)
Slovakia	Late	1.52	3.01	0.02		2.92 (17)	1.43 (9)
Slovenia	Early	1.70	6.78	0.00		9.11 (14)	2.81 (14)
Spain	Late	0.51	1.35	0.03		4.13 (12)	0.21 (12)
Sweden	Early	1.20	0.51	0.00		7.65 (14)	0.60 (14)
Switzerland	Early	1.12	1.31	0.03		–	–
Tajikistan	None	–	0.09	0.06		–	–
The former Yugoslav Republic of Macedonia	None	0.92	3.33	0.02		2.30 (4)	0.42 (1)
Turkey	Late	0.08	0.58	0.07		–	–
Turkmenistan	None	–	0.65	0.65		–	–
Ukraine	None	–	1.54	1.97		–	–
United Kingdom	Early	1.92	1.03	0.00		18.36 (11)	1.16 (11)
Uzbekistan	None	–	1.45	1.75		0.46 (2)	0.03 (1)
All	NA	1.20	3.07	0.74		7.76 (17)	1.03 (17)

NA: not applicable.

^a^ Countries that belong to the European Region of WHO.

^b^ Early: ban adopted by 2000; Late: ban adopted 2001–2013; None: no ban.

^c^ Values below 0.05 were given the value 0.00.

^d^ Time period 1994–2010.

^e^ *International classification of diseases*, 10th Revision, C45.

^f^ *International classification of diseases*, 10th Revision, J61.

For three consecutive periods Kazakhstan and the Russian Federation reported very high or high levels of asbestos use, while Kyrgyzstan, Ukraine and Uzbekistan recorded such levels for the two latter periods, and 11 countries did so for the two earlier periods.

Deaths due to mesothelioma were reported by 36 countries during 3–17 years and by one country during 1–2 years, while 16 countries did not report at all. Deaths caused by asbestosis were recorded by 26 countries during 3–17 years, by seven countries during 1–2 years and 20 countries did not provide any reports. Among the 36 countries that recorded mesothelioma mortality for three years or more, 21 countries recorded high age-adjusted mortality rates led by Iceland, followed by Malta and the United Kingdom of Great Britain and Northern Ireland. Among the 26 countries that recorded asbestosis mortality for three years or more, 12 recorded high age-adjusted mortality rates, led by Malta, followed by Slovenia and Finland.

During 1920–1970, 1971–2000 and 2001–2012, Europe used 31.2, 66.5 and 7.8 million metric tonnes of asbestos, respectively, accounting for 48%, 58% and 31% of the global use, respectively (Table 2). Europe recorded 71 686 deaths from mesothelioma (averaging 6786 deaths annually) corresponding to 56% of the global burden of such disease, and 5732 deaths from asbestosis (averaging 542 deaths annually) corresponding to 41% of the global asbestosis cases. Another 28 762 deaths were associated with mesothelioma, including deaths recorded as malignant neoplasm of the pleura in the WHO mortality database and those identified from scientific articles.^27^^–^^30^ In total, Europe registered 106 180 asbestos-related disease deaths, accounting for 60% of the global burden. Europe also had higher age-adjusted mortality rates for mesothelioma (7.8 versus 5.2 deaths/million persons/year) and asbestosis (1.0 versus 0.8 deaths/million persons/year) than the worldwide average.

**Table 2:** Asbestos use and related diseases, policies and health system rankings in Europe^a^ and worldwide, 1920–2012

Variable	Europe^a^				World (*n* = 194)^b^	Europe as % of the world
	Status of asbestos ban^c^			All (*n* = 53)		
	Early (*n* = 17)	Late (*n* = 17)	None (*n* = 19)			
**Population,** million people (%)	392 (43.2)	224 (24.6)	292 (32.1)	908 (100)	6974	13.0
**Asbestos use**						
Cumulative asbestos use, million metric tons (%)						
1920–1970	17.5 (56.2)	2.9 (9.2)	10.8 (34.6)	31.2 (100)	65.4^d^	47.7
1971–2000	17.2 (25.9)	6.6 (9.9)	42.7 (64.2)	66.5 (100)	113.8^e^	58.4
2001–2012	< 0.1 (0.3)	0.2 (2.5)	7.6 (97.2)	7.8 (100)	24.9^f^	31.4
Per capita asbestos use, kg/capita/year						
1920–1970	1.2	0.5	1.8	1.2	0.64	NA
1971–2000	1.6	1.2	8.0	3.1	0.87	NA
2001–2012	< 0.1	0.1	2.2	0.7	0.33	NA
**Asbestos-related disease**						
Mesothelioma (ICD-10, C45)						
Deaths, no. (no. of countries)	64 156 (16)	7407 (15)	123 (6)	71 686	128 635 (95)	55.7
Deaths, annual average (no. of years)	6270 (16)	590 (16)	19 (14)	6876	11 957	NA
AAMR, per million persons	9.4	3.7	3.2	7.8	5.2	NA
Mesothelioma, other data source^g^						
Deaths, no. (no. of countries)	26 885 (15)	1617 (10)	260 (3)	28 762	34 130 (53)^h^	87.3
Asbestosis (ICD-10, J61)						
Deaths, no. (no. of countries)	5385 (16)	339 (13)	8 (4)	5732	13 943 (60)	41.1
Deaths, annual average (no. of years)	493 (16)	43 (16)	5 (7)	542	1330	NA
AAMR, per million persons	0.8	0.9	1.5	1.0	0.8	NA
Asbestos-related diseases deaths, total no. (no. of countries)	96 426 (17)	9363 (16)	391 (8)	106 180	176 708 (105)^h^	60.1
**Related policies and health systems**						
Countries ratifying ILO Asbestos Convention, no. (% in country group)	9 (52.9)	6 (35.3)	5 (26.3)	20 (37.7)	35 (18.9)^i^	NA (NA)
Countries in top tertile of world ranking for health systems,^j^ no. (% in country group)	17 (100.00)	9 (52.9)	5 (26.3)	33 (62.3)	54 (27.8)	NA (NA)

AAMR: age-adjusted mortality rate; ICD*: International classification of diseases*; ILO: International Labour Organization; NA: not applicable.

^a^ Countries that belong to the European Region of WHO.

^b^ Number of countries in the world was based on the WHO definition.

^c^ Early: ban adopted by 2000; Late: ban adopted 2001–2013, None: no ban.

^d^ Based on 88 countries.

^e^ Based on 138 countries.

^f^ Based on 157 countries.

^g^ Malignant neoplasm of pleura (ICD-9, 163) in the WHO mortality database and published articles identified via PubMed of other source of national data [China (Hong Kong Special Administrative Region and Taiwan), Switzerland and Viet Nam]. For Switzerland, we estimated the values from a figure in Swiss Accident Insurance Institution (SUVA), Medical Information, No.78, p.65.

^h^ Taiwan, China, was counted as a single entity here, but it was not included in the 194 countries defined by WHO.

^i^ Number of ILO Member States is 185.

^j^ The ranking was obtained from the World Health Organization.^24^

Mesothelioma deaths were reported to the WHO mortality database by 16/17 (94%) early-ban countries, 15/17 (88%) late-ban countries and 6/19 (32%) no-ban countries. Of the 71 686 mesothelioma deaths throughout Europe, 64 156 (89%), 7407 (10%) and 123 (< 1%) were in the early-ban, late-ban and no-ban groups, respectively. Countries in the early-ban, late-ban and no-ban groups reporting mesothelioma deaths had age-adjusted mortality rates (crude mortality rates) of 9.4 (16.5), 3.7 (4.7) and 3.2 (0.6) deaths/million persons/year, respectively. Asbestosis deaths were reported by 16/17 (94%) early-ban countries, 13/17 (76%) late-ban countries, and 4/19 (21%) no-ban countries. Of the 5732 asbestosis deaths throughout Europe, 5385 (94%), 339 (6%) and 8 (< 1%) were in the early-ban, late-ban and no-ban groups, respectively. Countries in the early-ban, late-ban and no-ban groups that reported asbestosis had age-adjusted mortality rates (crude mortality rate) of 0.8 (1.4), 0.9 (0.3) and 1.5 (0.2) deaths/million persons/year, respectively.

The ratification rates of the ILO Asbestos Convention were higher in Europe than worldwide (38% versus 19%). Also the quality ranking for the health systems was higher in Europe (62% versus 28%). Within Europe, the convention was ratified by 53% (9/17), 35% (6/17) and 26% (5/19) countries in the early-ban, late-ban and no-ban groups, respectively. Higher-ranking health systems were found in 100% (17/17), 53% (9/17) and 26% (5/19) of these groups, respectively (Table 2).

## Discussion

This descriptive analysis of data in the WHO mortality database shows that 56% of all mesothelioma deaths and 41% of all asbestosis deaths recorded worldwide occurred in Europe, which accommodates 13% of the world’s population. Combining these data with those from other sources showed that Europe accounted for 60% of the reported global deaths from asbestos-related diseases, excluding asbestos-induced lung cancer. During the periods 1920–1970 and 1971–2000, Europe used 48% and 58%, respectively, of all asbestos traded throughout the world. Europe can thus be characterized as the historical global centre of asbestos use and the current global centre of reported asbestos-related diseases.

The three different ban groups had comparable population sizes but showed wide differences in the absolute numbers of asbestos-related disease deaths. The early-ban group reported the highest burden of asbestos related disease, while the no-ban group recorded the lowest. This could reflect differences in the reporting of asbestos-related diseases, as the majority of early-ban and late-ban countries reported asbestos-related disease data, whereas most of the no-ban countries did not. The three groups also differed in the quality rankings of their health systems,^24^ prompting us to speculate that gaps may exist in the level of medical expertise and resources required to diagnose and report asbestos-related diseases.

Almost all countries (14/17) that used asbestos at very high or high levels during 1920–1970 also demonstrated high mortality rates from mesothelioma and/or asbestosis. Kazakhstan and the Russian Federation did not report such data to WHO and no other comparable data could be identified. Switzerland did not report data to the WHO, but a substantial mesothelioma burden was found in a scientific article reporting national data.^28^ Israel constituted the only identified exception to the relationship between asbestos use and asbestos-related diseases.

We have shown earlier that the level of asbestos use correlates with the subsequent asbestos-related disease burden.^18^ The asbestos-related disease burdens observed in the early-ban and late-ban countries are thus likely to be proportional to their levels of earlier asbestos use. The lower asbestos-related disease mortality currently being recorded by the no-ban group – despite higher levels of earlier asbestos use – is based on sparse data and likely reflects underdiagnosis and underreporting.

Asbestos use can be influenced by national policies. Therefore, we assessed the ratification status of the ILO Asbestos Convention.^23^ The ratification rate was highest in the early-ban group and lowest in the no-ban group, suggesting a possible influence. It is also plausible that ratification may not have influenced use, but rather reduced asbestos exposure. A directive of the European Union (EU) mandated that all member states ban asbestos from 2005.^36^^,^^37^ However, some individual EU countries began adopting bans as early as the 1980s. The EU countries thus achieved zero use at different time points. In contrast, two no-ban countries, Kazakhstan and the Russian Federation have both used and mined asbestos^5^^,^^6^ in recent years, at approximately 930 000 and 280 000 metric tonnes per year, respectively (RL Virta, United States Geological Survey, personal communication). Economic incentives in these countries may encourage domestic and international asbestos use.

Between 2001 and 2012, Europe used 7.8 million metric tonnes of asbestos. This share (31% of global use) is still disproportionately high relative to the population of this region. However, the absolute use declined from 3.1 kg/capita/year during 1971–2000 to 0.7 kg/capita/year during 2001–2012. Also the level of use varies considerably by group. The early-ban and late-ban groups reduced their average use to less or equal to 0.1 kg/capita/year, respectively, whereas the no-ban group continued to use an average of 2.2 kg/capita/year. In the early-ban group the use varied between 0 and 0.19 kg/capita/year, which might be variable according to the extent of national exemptions. These are considerable reductions from the previously high levels of use observed between 1920 and 2000 in the early-ban group and between 1971 and 2000 in the late-ban group. In contrast the no-ban group recorded very high and high levels of asbestos use throughout the timeframe studied. The six countries with very high and high levels of use in the present century were all no-ban countries. Hence, although asbestos use was historically widespread and substantial across most of Europe, more recent use has been concentrated in the no-ban countries. We therefore speculate that the future burden of asbestos-related diseases will likely shift from the early-ban and late-ban countries towards the no-ban countries.

We previously used similar methods to analyse asbestos use and asbestos-related diseases in Asia.^35^ Although the previous and present findings can be roughly compared, some caution should be exercised. The earlier study adopted the United Nations Statistical Division definition of Asia, whereas the present study adopted the WHO definition of Europe, resulting in an overlap of 11 countries, including one early-ban country (Israel), two late-ban countries (Cyprus and Turkey), and eight no-ban countries (Armenia, Azerbaijan, Georgia, Kazakhstan, Kyrgyzstan, Tajikistan, Turkmenistan and Uzbekistan). Also, the final years analysed for asbestos use and asbestos-related diseases in the earlier study were 2007 and 2008, respectively.

A strength of this study is the use of quantitative data from public databases to describe the situations in many countries. There were limitations, however, in the representation and comparability of the analysed data. For example, the data on use of asbestos were extrapolated for several countries that lacked specific data due to political transitions. In addition, data on the use of imported asbestos-containing products were not available. Moreover, as asbestos-related diseases are generally rare and difficult to diagnose, serious bias could have been introduced by countries having limited experience with asbestos-related diseases.

In conclusion, Europe currently carries the majority of the global asbestos-related disease burden as a consequence of heavy asbestos use during earlier decades. For countries that have stopped using asbestos, their asbestos-related disease burden will most likely decrease. In contrast, countries that still have not banned asbestos are likely to have a substantial burden of asbestos-related disease in the future due to their past and current high levels of asbestos use. As attempts to reduce exposure without a concurrent reduction in overall use are insufficient to control risk,^16^^,^^38^ asbestos bans should be in place in all countries to eliminate asbestos-related diseases.
